# Annotation and evaluation of base editing outcomes in multiple cell types using CRISPRbase

**DOI:** 10.1093/nar/gkac967

**Published:** 2022-11-09

**Authors:** Jibiao Fan, Leisheng Shi, Qi Liu, Zhipeng Zhu, Fan Wang, Runxian Song, Jimeng Su, Degui Zhou, Xiao Chen, Kailong Li, Lixiang Xue, Lichao Sun, Fengbiao Mao

**Affiliations:** Institute of Medical Innovation and Research, Peking University Third Hospital, Beijing 100191, China; College of Animal Science and Technology, Yangzhou University, Yangzhou, Jiangsu Province 225009, China; Institute of Medical Innovation and Research, Peking University Third Hospital, Beijing 100191, China; Cancer Center, Peking University Third Hospital, Beijing 100191, China; Rice Research Institute, Guangdong Academy of Agricultural Sciences, Guangzhou 510640, China; Guangdong Key Laboratory of New Technology in Rice Breeding, Guangzhou 510640, China; Guangdong Rice Engineering Laboratory, Guangzhou 510640, China; Institute of Medical Innovation and Research, Peking University Third Hospital, Beijing 100191, China; Cancer Center, Peking University Third Hospital, Beijing 100191, China; College of Animal Science and Technology, Yangzhou University, Yangzhou, Jiangsu Province 225009, China; Rice Research Institute, Guangdong Academy of Agricultural Sciences, Guangzhou 510640, China; State Key Laboratory of Tree Genetics and Breeding, Forestry College, Northeast Forestry University, Harbin 150040, China; College of Animal Science and Technology, Yangzhou University, Yangzhou, Jiangsu Province 225009, China; Rice Research Institute, Guangdong Academy of Agricultural Sciences, Guangzhou 510640, China; Guangdong Key Laboratory of New Technology in Rice Breeding, Guangzhou 510640, China; Guangdong Rice Engineering Laboratory, Guangzhou 510640, China; Laboratory of Marine Protozoan Biodiversity & Evolution, Marine College, Shandong University, Weihai 264209, China; Department of Biochemistry and Biophysics, School of Basic Medical Sciences, Peking University Health Science Center, Beijing 100191, China; Institute of Medical Innovation and Research, Peking University Third Hospital, Beijing 100191, China; Cancer Center, Peking University Third Hospital, Beijing 100191, China; State Key Laboratory of Molecular Oncology, National Cancer Center/National Clinical Research Center for Cancer/Cancer Hospital, Chinese Academy of Medical Sciences and Peking Union Medical College, Beijing 100021, China; Institute of Medical Innovation and Research, Peking University Third Hospital, Beijing 100191, China; Cancer Center, Peking University Third Hospital, Beijing 100191, China

## Abstract

CRISPR-Cas base editing (BE) system is a powerful tool to expand the scope and efficiency of genome editing with single-nucleotide resolution. The editing efficiency, product purity, and off-target effect differ among various BE systems. Herein, we developed CRISPRbase (http://crisprbase.maolab.org), by integrating 1 252 935 records of base editing outcomes in more than 50 cell types from 17 species. CRISPRbase helps to evaluate the putative editing precision of different BE systems by integrating multiple annotations, functional predictions and a blasting system for single-guide RNA sequences. We systematically assessed the editing window, editing efficiency and product purity of various BE systems. Intensive efforts were focused on increasing the editing efficiency and product purity of base editors since the byproduct could be detrimental in certain applications. Remarkably, more than half of cancer-related off-target mutations were non-synonymous and extremely damaging to protein functions in most common tumor types. Luckily, most of these cancer-related mutations were passenger mutations (4840/5703, 84.87%) rather than cancer driver mutations (863/5703, 15.13%), indicating a weak effect of off-target mutations on carcinogenesis. In summary, CRISPRbase is a powerful and convenient tool to study the outcomes of different base editors and help researchers choose appropriate BE designs for functional studies.

## INTRODUCTION

Recently, the introduction of Clustered Regularly Interspaced Short Palindromic Repeats (CRISPR) system allowed us to investigate the potential clinical value of genetic alterations in the human genome with clinical relevance. CRISPR-Cas-induced double-strand breaks (DSBs) on DNA can lead to unintended chromosomal alterations or elicit an unwanted DNA damage response. Subsequently, DSBs are typically repaired through one of two competing endogenous repair pathways in the cells, non-homologous end joining (NHEJ) or homology-directed recombination (HDR) (1). NHEJ-repaired DSBs result in insertions/deletions (indels) at the target region, often leading to a frameshift and gene knockout. HDR can be used to introduce desired and precise sequence edits into the genome while HDR efficiencies vary among cell types and are only active during certain cell cycle phases (2).

Approximately half of the known pathogenic genetic variants are due to single-nucleotide variants (SNVs), and most of the observed SNVs lack clinical interpretations (3). Thus, base editing (BE) technology is developed to address the challenge and can catalyze highly efficient base transition without inducing DSB (4). CRISPR-Cas base-editing technology enables targeted nucleotide alterations and is being increasingly used for research and potential therapeutic applications (5,6). BE at single-base resolution has already been successfully performed in plants, yeast, and human cells (4,7–9). To avoid generating DSBs, diverse modified Cas proteins have been employed in BE systems, including nuclease-dead Cas9 (dCas9) and Cas9 nickase (nCas9), and other modified nucleases, such as d/nCas12a, d/nCas12b, d/nCasX and d/nCas13 (10). A growing number of studies have indicated that BE system is a powerful tool to manipulate genetic base editing *in vitro* and *in vivo* (4,9,11). Consequently, the CRISPR/Cas BE data is increasing rapidly, posing a challenge for researchers to access all related information on BE outcomes.

In contrast, deamination with BE systems at undesired RNA or DNA sites can cause unknown off-target effects (12). Although great efforts have been made to minimize the off-target effect by engineering deaminases or Cas9 proteins (13–16), the off-target effect of base editing is still the major safety concern for its application to gene therapy and the production of genetically modified animals and plants (16,17). The off-target effect remains unknown and differs among various BE systems. Hence, an integrated resource with an analysis function is urgently needed for comprehensive annotation and evaluation of the on-target efficiency and the off-target effect among various BE systems.

**Figure 1.**
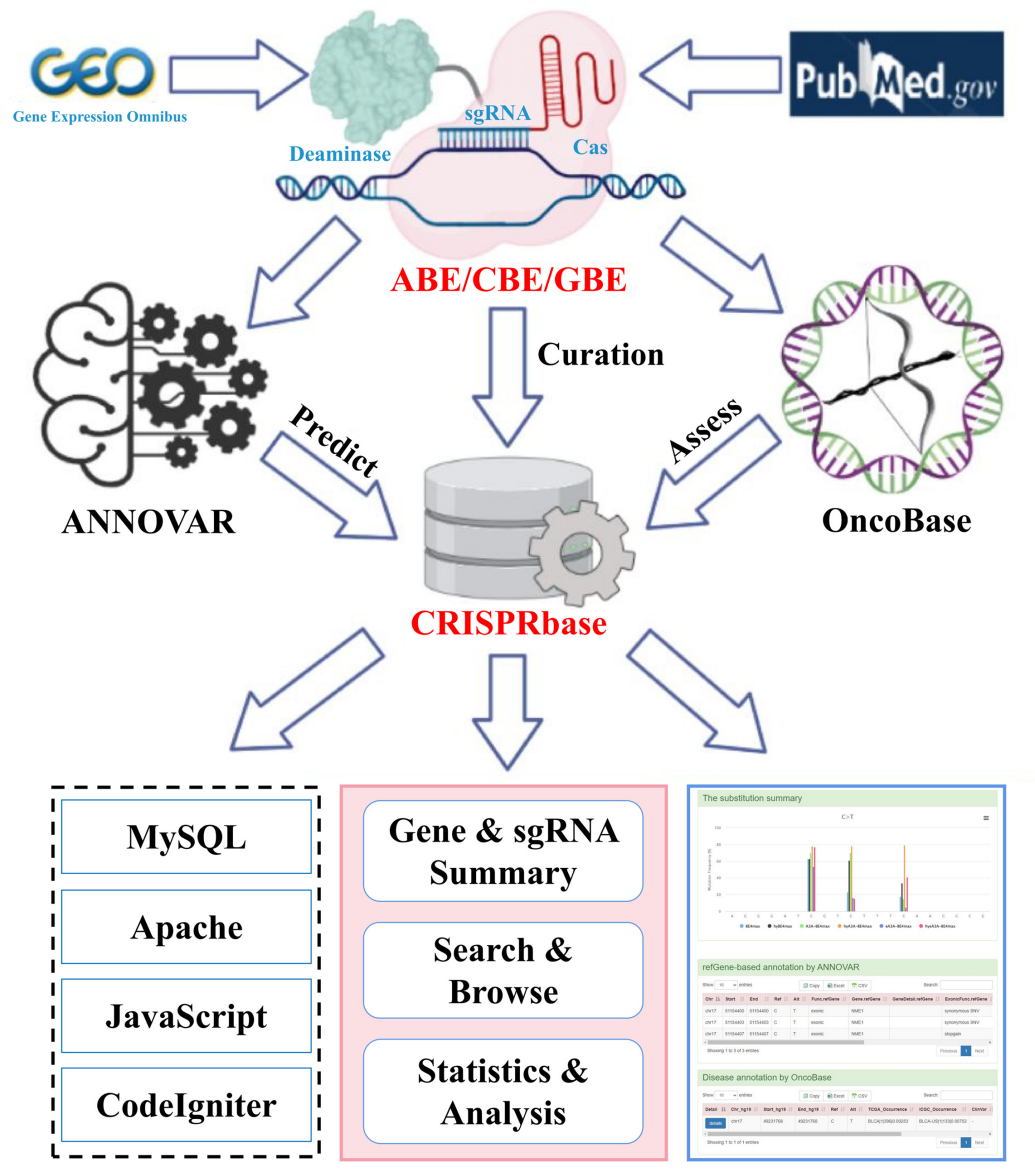
Workflow of CRISPRbase construction. CRISPRbase is a comprehensive database curating the editing outcome and evaluating off-target effects of base editors on various cell types and tissues in dozens of species. We collected >1.2 million records of base editing outcomes and annotated these outcomes with functional and oncogenic effects of mutations using ANNOVAR and OncoBase, respectively. Furthermore, our database comprises a search tool which allows searching for deposited sgRNA sequences with ≤4 mismatches to the searched sequence. Academic users can freely search, browse, and download related data and obtain analytical results through the web interface.

Herein, we developed CRISPRbase, a novel platform that integrated comprehensive information of 1 252 935 records of BE outcomes in 54 cell types from 17 species (Figure 1). CRISPRbase provides putative editing precision and off-target effect of different BE systems by incorporating multiple annotations including ‘organisms’, ‘genome build’, ‘chromosome’, ‘position’, ‘sgRNA sequences’, ‘PAM sequences’, ‘strand direction’, ‘mutant’, ‘editing systems’, ‘cell/tissue name’, ‘Cas variant’ and ‘mutation frequency’. Moreover, we predicted the functional effects of off-target mutations induced by BE systems using ANNOVAR software (18) and annotated the oncogenic effects of off-target mutations using OncoBase (19). Furthermore, CRISPRbase was designed to provide the gene expression changes before and after BE induced by different BE systems. Most importantly, users could search the sgRNA sequence of interest by blasting against the deposited sgRNA sequences to infer BE ability with similar BE systems. CRISPRbase is not only a convenient database but also a powerful tool to study the outcome of different base editors and help researchers select appropriate BE designs for functional studies. We are dedicated to maintaining CRISPRbase and keeping the resources updated in the future.

## MATERIALS AND METHODS

### Data collection

Data were collected manually to construct the CRISPRbase from the published papers, and data lacking precise position or sgRNA information was filtered out. CRISPRbase comprised >1 million sgRNAs collected from 186 published papers from PubMed (Supplemental Table S1) and included the following main information: (i) Organisms that have been edited by the base editors; (ii) sgRNA information including sequences, localizations, and directions on reference genomes; (iii) names of the target genes; (iv) protospacer adjacent motif (PAM) sequences and Cas protein variants; (v) base editor types including adenine base editor (ABE), adenine and cytosine base editor (ACBE), cytosine base editor (CBE), cytosine or adenine base editor (CBE_ABE), cytosine to guanine base editor (CGBE), plant dual‐base editor (pDuBE), plant base editor (PBE), and PmCDA1-based cytosine-to-thymine base editor (PCBE); (vi) library types; (vii) editing efficacy; (viii) expression change of target genes before and after editing; (ix) cell/tissue names; (x) screen type and treatment.

### Target gene expression

First, mapped reads of the target genes were normalized to log RPM (reads per million mapped reads) as follows:

$$\begin{equation*}\log\,{\rm RPM} = {{\rm log}_2}\;\left( {\frac{{{\rm reads}\;{\rm per}\;{\rm guide}}}{{{\rm total}\;{\rm reads}\;{\rm per}\;{\rm condition}}} \times 1\,000\,000 + 1} \right)\end{equation*}$$

Then, the logarithmic fold change before and after base editing (logFCBE) was calculated using the following formula:

$$\begin{equation*}{\rm{log\,FCBE\;}} = {\log_2}\;\left( {\frac{{\log\,{{\rm RPM}_{{\rm after}\;{\rm editing}}} + 1}}{{\log\,{{\rm RPM}_{{\rm before}\;{\rm editing}}} + 1}}} \right)\end{equation*}$$

### Mapping sgRNAs to the latest genomes

Since the reference genomes of sgRNAs from different sources of the same species are inconsistent, we uniformly mapped sgRNA sequences to the latest reference genomes using Bowtie2 with parameters ‘bowtie2 –very-sensitive –end-to-end -p 16 -x genome_index -U input.fa -f -S input.fa.sam’ (20). For unaligned sgRNAs, we checked whether they were derived from mutant cells and converted them to wild type to get a genomic position. All the reference genomes were downloaded from the NCBI RefSeq database (https://www.ncbi.nlm.nih.gov/refseq/).

### Functional prediction with ANNOVAR

To identify whether mutations induced by the off-target effect alter protein coding and affect the amino acid sequence, we used gene-based annotation and functionally annotated genetic variants detected from diverse genomes with parameters ‘annovar/table_annovar.pl –buildver genome_build –remove –otherinfo –protocol refGene –operation g variant.txt annovar/db’ by using ANNOVAR software (18).

### Cancer and disease annotation with OncoBase

OncoBase was used to annotate BE-induced mutations by exploring their regulatory roles in chromatin interactions between target genes and regulatory elements in multiple cancer types (19). The included annotations from OncoBase in CRISPRbase were TCGC occurrence, ClinVar diseases, GWAS, MGI Phenotype and cancer risk assessment from HGMD, and an external link to OncoBase to get more detailed annotation.

### Motif analysis and mutation rate analysis

For the motif analysis, we collected the mutation information of all sites within a 20 bp window from the start site of targets for each sgRNA. For a site without mutations, all four possible nucleotides (A, T, C and G) shared equal weight. For the mutated sites, the two allele nucleotides shared all the weight. The weight matrices of all single-base editing systems were transformed to position weight matrix (PWM), which was then used to draw the motif logos using R package seqLogo (21). Meanwhile, for the 20 bp windows of each BE system, the mutation rates of all mutation sites were collected and a boxplot plot was used to show the mutation rate distribution for each site in the editing window. A heatmap was used to show the mutation rate distribution for the window among different BE systems based on the mean value of the mutation rate for each site. The editing efficiencies of all BE systems were collected and a boxplot was used to show the efficiency distribution among all BE systems.

### Association between gene expression change and mutation frequency

Since the expression change of each gene may be caused by multiple mutations located in the gene, we selected the mutations with the highest mutation frequency to investigate the potential relationship between the mutation efficiency and the change in gene expression. Pearson's correlation coefficient was calculated using R.

### Analysis of the off-target mutation effect

Canonically, the edit window of the base editing system was 4th to 8th bases on the sgRNA-targeting sequence. If BE-induced mutations occurred outside of the edit window or were unexpected nucleotide mutations (e.g. CBE induced A-G mutation), we defined these mutations as off-target mutations. Since we wondered if any of these off-target mutations were oncogenic mutations, they were further overlapped with cancer-related somatic mutations that occur in 36 tumor types obtained from OncoBase (Supplemental Table S2) (19). We then classified these cancer-related off-target mutations as ‘nonsynonymous’ or ‘stopgain’ or ‘splicing’, ‘synonymous’ and ‘unknown’ using VarCards (22). Furthermore, these ‘nonsynonymous’ and ‘stopgain’ mutations were predicted as extreme/damaging or benign mutations affecting protein function using VarCards (22) and classified into cancer driver or passenger mutations using AI-Driver with a cutoff of 0.95 (23). In detail, we divided extreme mutations into loss-of-function or nonsynonymous mutations according to a damaging score >0.5 and an allele frequency of <0.0001 based on gnomAD (https://gnomad.broadinstitute.org/). The damaging score was defined as the proportion in 23 algorithms predicted to be deleterious while the damaging score of loss-of-function mutation was deemed to be 1.

## RESULTS

### Data statistics and website interface

**Figure 2.**
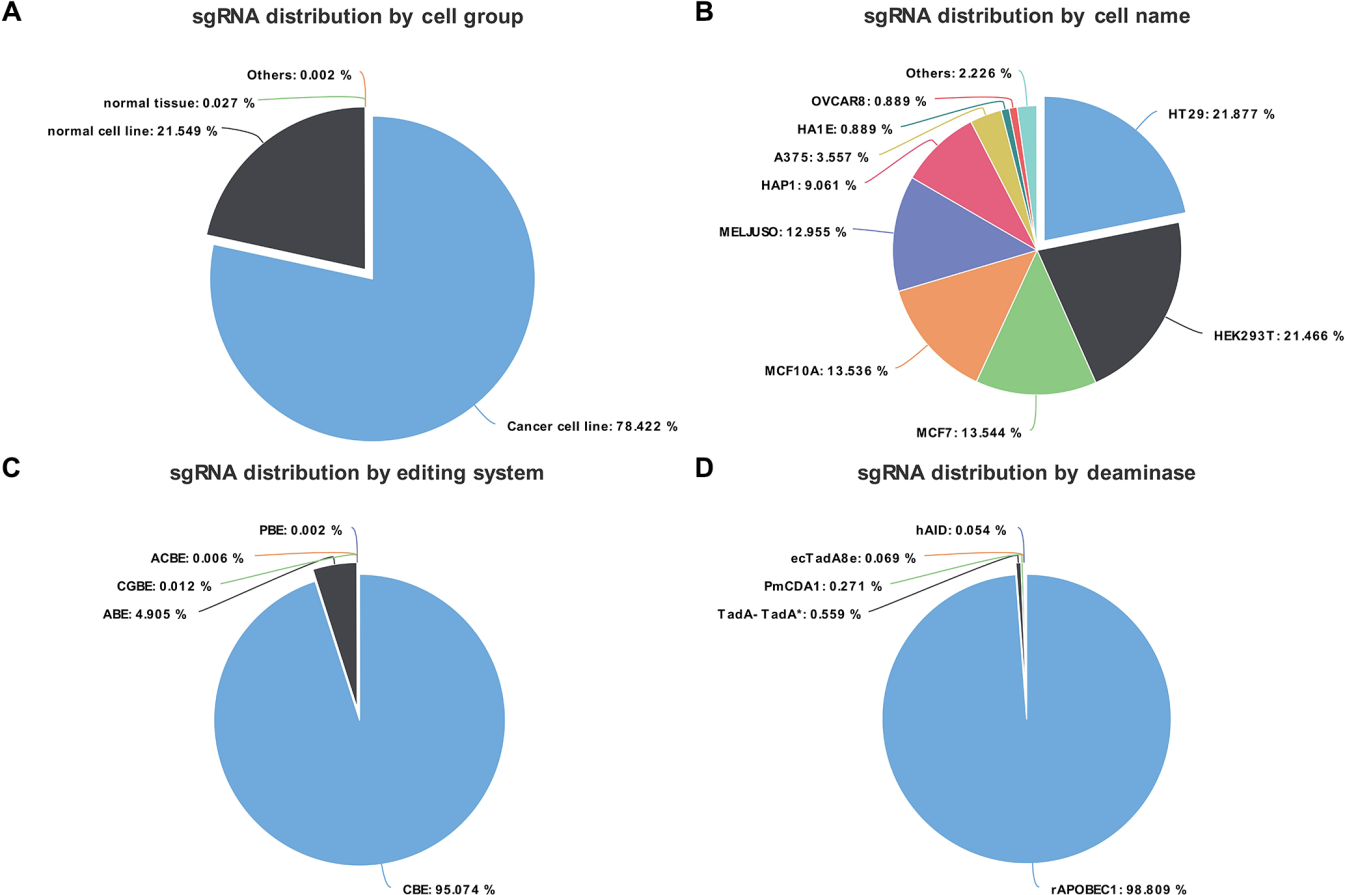
sgRNA distribution on CRISPRbase. (**A**) 982 586, 269 995 and 344 sgRNAs were distributed in cancer cell lines, normal cell lines, and normal tissues, respectively. (**B**) 274 104, 268 959, 169 696, 169 602, 162 314, 113 527 and 445 64 sgRNAs were collected from HT29, HEK293T, MCF7, MCF10A, MELJUSO, HAP1, A375 and other cells, respectively. (**C**) 1 191 225, 61 460, 156, 69, 20 and 10 sgRNAs were distributed in editing systems of CBE, ABE, CGBE, ACBE, PBE, and PCBE, respectively. (**D**) sgRNAs were sorted by deaminase types including 989 325 rAPOBEC1, 5 592 TadA-TadA*, 2 711 PmCDA1, 694 ecTadA8e and others.

CRISPRbase compiled available BE outcomes (*n* = 1 252 935) from public resources and comprehensively interpreted those in various cancer cell lines (n = 982 586), normal cell lines (*n* = 269 995), and normal tissues (n = 344) (Figure 2A). We collected 274 104 BEs for HT29 cells, 268 959 for HEK293T cells, 169 696 for MCF7 cells, 169 602 for MCF10A cells, 162 314 for MELJUSO cells, 113 527 for HAP1 cells and 445 64 for A375 cells, as well as others (Figure 2B). Our collection covered 1 191 225 CBEs, 61 460 ABEs, 156 CGBEs, 69 ACBEs, 20 PBEs and 10 PCBEs (Figure 2C). These sgRNAs were sorted by deaminase types including 989 325 rAPOBEC1, 5592 TadA-TadA*, 2 711 PmCDA1, 694 ecTadA8e and others (Figure 2D).

CRISPRbase comprised Home, Search, Analysis, Submission, Download, Stats, Manual and Contact modules. A brief introduction of CRISPRbase and the workflow was given in the Home module. Data were integrated into the Search module of CRISPRbase and could be searched by gene symbol and genomic region with filtering options based on the organism, editing system type, cell name and cell group. Moreover, we provided a function in the Analysis module for users to blast their sgRNA sequence of interest to search curated sgRNA sequences with a maximum of four base mismatches. In the Stats module, distributions of BE records under various criteria, including organism, editing system type, cell group, and cell name, were exhibited in pie charts. In the Manual module, steps of how to use CRISPRbase were illustrated. In the Contact module, the e-mail addresses and research fields of related authors were described in detail.

### Editing efficiency and product purity of base editors

**Figure 3.**
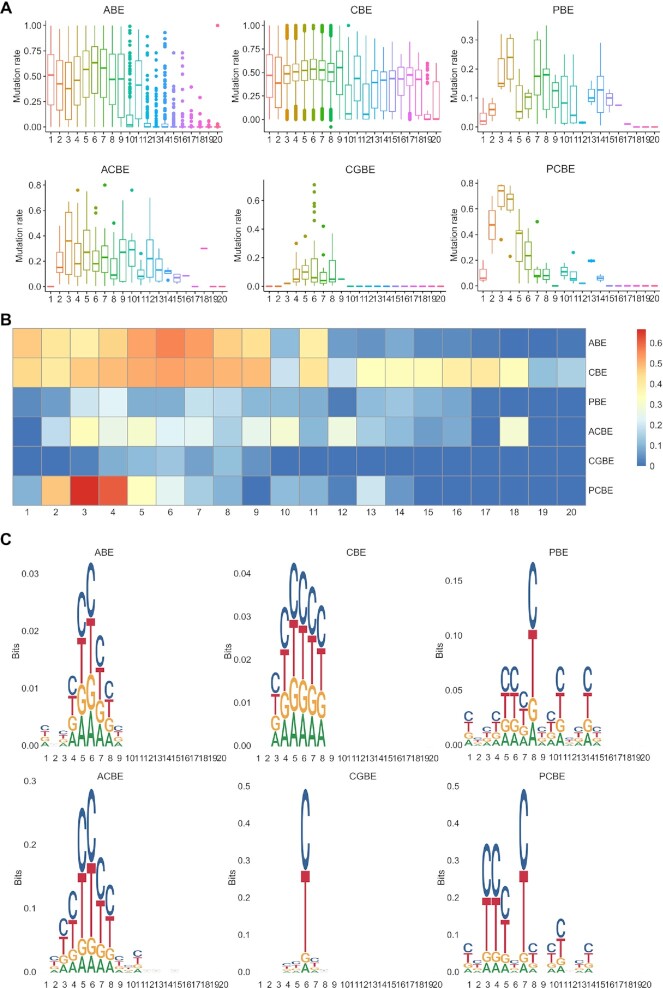
The features of CRISPRbase. (**A**) Boxplot of the editing efficiency of different editing systems at different positions on target sequences. (**B**) Heatmap of the editing efficiency of the different editing systems at different positions on target sequences. (**C**) Sequence motifs of the different editing systems. The editing window was within position 1–15 in different editing systems, and the stable editing efficiency was 25–75% for CBE and ABE (A, B), and specific points were indicated in the editing systems (C).

We measured the overall editing window, efficiency, and product purity of six kinds of CRISPR-Cas base editors within a 20-bp window (Figure 3A-C). CBE (position 1–20) exhibited the widest editing window among these BE systems (Figure 3A, B). The editing window of ABE (position 1–11), PBE (position 1–15), PCBE (position 1–14), and ACBE (position 2–15) was slightly narrower than that of CBE and was slightly wider than that of CGBE (position 4–8). CBE exhibited stable editing efficiency (25–75%) within the 20 bp window (Figure 3A, B). ABE presented stable editing efficiency (25–75%) within positions 1–11. The main editing efficiencies of PBE (0–30%) and ACBE (0–60%) were mainly focused on position 2–15, but the efficiencies of other positions were almost equal to 0. Moreover, the editing efficiencies of CGBE were under 20% for almost all the positions. Noticeably, the editing efficiency of PCBE differed greatly in different positions, with the editing efficiency almost being 80% for position 3–4, but 20–60% for positions 2 and 5–6, and < 20% for other positions. For product purity (Figure 3C), CGBE introduced point mutations with a high degree of product purity at a specific site (position 6). Although the editing windows of PBE, ACBE, and PCBE were relatively wider, only specific point mutations were cleanly introduced for PBE (position 8), ACBE (positions 5, 6), and PCBE (positions 3–4, 7). Notably, the product purities of ABE and CBE were relatively low at all editing points, indicating undesirable outcomes among these BE systems.

### Gene expression change and off-targeting effect induced by BE

**Figure 4.**
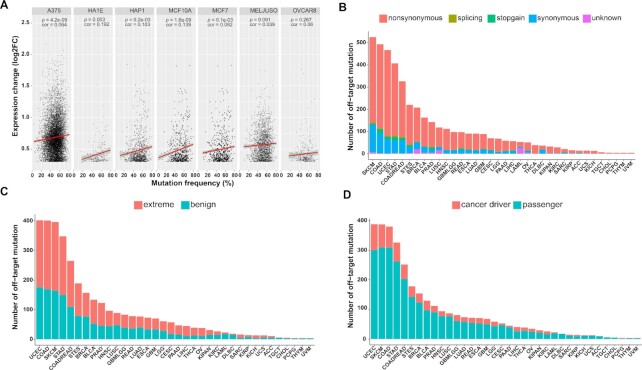
Profiling of on-target and off-target effects in different cell types and tissues. (**A**) Single-base mutations were correlated with gene expression in A375, HA1E, HAP1, MCF10A and MCF7 cells. The analysis was conducted via a two-tailed test based on Pearson's product-moment correlation coefficient. (**B**) More than half of off-target mutations were non-synonymous. (**C**) The number of extreme and benign damage mutations induced via off-target mutations among different cancer types. More than half of non-synonymous mutations were extreme mutations in 24 tumor types. (**D**) The number of cancer driver mutations was significantly lower than that of passenger mutations within non-synonymous mutations.

We selected seven kinds of human cell lines with gene expression information to investigate the associations between gene expression change and dominant mutation frequency (Figure 4A). We found that expression changes and mutation frequency displayed no significant association in MELJUSO (cor = 0.039; *P* = 0.091) and OVCAR8 (cor = 0.06; *P* = 0.267) cell lines. Although slight positive correlations were observed in A375 (cor = 0.064; *P* = 4.2e–09), HA1E (cor = 0.192; *P* = 0.03), HAP1 (cor = 0.103; *P* = 0.2e-03), MCF10A (cor = 0.139; *P* = 1.8e–09), and MCF7 cells (cor = 0.08, *P* = 0.1e–03), our observations indicated that single-base mutations induced by BE systems had a weak effect on gene expression changes.

We further integrated cancer somatic mutations in 36 common tumor types from OncoBase to assess the effect of off-target mutations on protein function (Figure 4B-D). We identified 64 894 off-target mutations induced by BEs, in which 525, 493, 467 and 407 mutations were related to skin cutaneous melanoma (SKCM), colon adenocarcinoma (COAD), uterine corpus endometrial carcinoma (UCEC), and stomach adenocarcinoma (STAD), respectively (Supplemental Table S3). We then depicted the types of these cancer-related off-target mutations and found that more than half of them were non-synonymous mutations, except for mutations related to acute myeloid leukemia (LAML) and lymphoid neoplasm diffuse large B-cell lymphoma (DLBC) (Figure 4B). We next assessed the percentages of extreme and benign mutations induced by non-synonymous and stop-gain mutations using VarCards (22) (Figure 4C). More than half of these non-synonymous mutations were extreme mutations in 24 tumor types, such as SKCM, COAD, UCEC and STAD, indicating that most of these cancer-related off-target mutations were extreme mutations related to most tumor types. However, the percentage of cancer-driver mutations in these non-synonymous mutations was significantly lower than that of passenger mutations in all 36 common tumor types (Figure 4D). Although our analysis was based on functional prediction using AI-Driver (23), it could preliminarily decipher a mild effect of off-target mutations on carcinogenesis.

## DISCUSSION

Herein, we developed the CRISPRbase platform to curate experimentally validated BE events, and evaluate the potential off-target effects using ANNOVAR and OncoBase. In this study, we found that the editing efficiency, product purity, and off-target effects differ among various BE systems. Although the editing windows of ABE and CBE were relatively wider, their editing efficiencies were higher than other BEs. The editing efficiency of CGBE was as low as 20% for almost all the positions though CGBE introduced point mutations with a high degree of product purity at one specific site (position 6). Thus, intensive efforts were focused on increasing the editing efficiency and product purity of base editors since the byproduct could be detrimental in certain applications. Only specific point mutations were introduced for PBE (position 8), ACBE (positions 5, 6), and PCBE. However, more experimental records are warranted to confirm the observations for CGBEs, ACBEs, PBEs, and PCBEs (positions 3–4, 7). Noticeably, more than half of cancer-related mutations induced by off-target effects were non-synonymous and could induce extremely damaging effects on protein function in most common tumor types. Luckily, most of these cancer-related mutations were passenger mutations (4840/5703, 84.87%) rather than cancer driver mutations (863/5703, 15.13%), indicating a mild effect of off-target mutations on carcinogenesis.

Currently, more tools are being developed to help design sgRNAs and predict the outcome of CRISPR BE. Hwang and colleagues developed two web tools for BE, named BE-Designer and BE-Analyzer (24). BE-Designer provides all possible base editor target sequences in a given input DNA sequence with useful information including potential off-target sites. BE-Analyzer assesses BE outcomes using next-generation sequencing (NGS) data and provides information about mutations in the form of a table and interactive graphs. Xie and colleagues published a versatile web-based tool to design guide RNAs for BE in plants (25). Siegner and colleagues developed PnB Designer, a web application to design prime and base editor guide RNAs for animals and plants (26).

Moreover, several machine learning and deep learning methods have been developed for in-silico prediction of BE outcomes based on training datasets of cytosine and adenine editors: BE-HIVE (13), an autoregressive neural network model; DeepCBE/DeepABE (27), a convolutional neural network model; FORECasT (28), a gradient boosted tree model and BE-DICT (29), an attention-based deep learning algorithm. However, the predicting accuracy for the editing efficiency of these tools is still not high enough, ranging from 0.49 to 0.72 (28). Thus, carefully curated reference records are urgently required to improve the model-based prediction or estimate the outcome of certain base editing designs. However, there is no comprehensive database to curate the outcome and evaluate the off-target effect of BEs across cell types and tissues.

As far as we know, CRISPRbase is the first database for CRISPR-mediated BE and has several advantages. It (i) provides comprehensive annotations of BE outcomes; (ii) is fitted with the prediction of BE off-target effects using ANNOVAR and OncoBase; (iii) has a blast system based on curated sgRNA sequences for better decision-making of BE design for new genes or locations.

In summary, CRISPRbase curates BE events in various cell types from multiple species and provides a system to search for similar sgRNA designs based on curated records. Some of the functions in our current database need further refinement and more effort has to be paid to several aspects including expanding the record number with the latest publications, embedding a sequence-based prediction model with a deep learning algorithm, and extending annotation terms of off-target effects. Like our previously developed platforms (19,23,30–36), we are dedicated to maintaining and improving CRISPRbase. We hope it will be a very useful platform for both researchers and the clinical community.

## DATA AVAILABILITY

The full tables of curated base editing records are available in the Download module of CRISPRbase (http://crisprbase.maolab.org).

## Supplementary Material

gkac967_Supplemental_Files
